# The transcriptome of *Mycobacterium tuberculosis* in a lipid-rich dormancy model through RNAseq analysis

**DOI:** 10.1038/s41598-017-17751-x

**Published:** 2017-12-15

**Authors:** Diana A. Aguilar-Ayala, Laurentijn Tilleman, Filip Van Nieuwerburgh, Dieter Deforce, Juan Carlos Palomino, Peter Vandamme, Jorge A. Gonzalez-Y-Merchand, Anandi Martin

**Affiliations:** 1 Laboratory of Microbiology, Faculty of Science, Ghent University, Gent, Belgium; 2 Laboratory of Molecular Microbiology, Escuela Nacional de Ciencias Biológicas, Instituto Politécnico Nacional, Mexico City, Mexico; 3 Laboratory of Pharmaceutical Biotechnology, Faculty of Pharmaceutical Sciences, Ghent University, Gent, Belgium; 4 Pôle of Medical Microbiology, Institute of Experimental and Clinical Research, Université Catholique de, Louvain, Brussels Belgium

## Abstract

Tuberculosis (TB) is currently the number one killer among infectious diseases worldwide. Lipids are abundant molecules during the infectious cycle of *Mycobacterium tuberculosis* (*Mtb*) and studies better mimicking its actual metabolic state during pathogenesis are needed. Though most studies have focused on the mycobacterial lipid metabolism under standard culture conditions, little is known about the transcriptome of *Mtb* in a lipid environment. Here we determined the transcriptome of *Mtb* H37Rv in a lipid-rich environment (cholesterol and fatty acid) under aerobic and hypoxic conditions, using RNAseq. Lipids significantly induced the expression of 368 genes. A main core lipid response was observed involving efflux systems, iron caption and sulfur reduction. In co-expression with ncRNAs and other genes discussed below, may act coordinately to prepare the machinery conferring drug tolerance and increasing a persistent population. Our findings could be useful to tag relevant pathways for the development of new drugs, vaccines and new strategies to control TB.

## Introduction

*Mycobacterium tuberculosis* (*Mtb*) remains a leading infectious agent since first described by Robert Koch as the cause of tuberculosis (TB) and is now the first killer among infectious diseases^1^. *Mtb* successfully disseminates based in part on its exceptional mechanisms of infection, survival, persistence and dormancy. Drug tolerance and drug resistance, metabolic reactivation from dormancy, and dissemination inside the host, all contribute to its successful pathogenesis and transmission. The World Health Organization declared TB as a global emergency in 1993 and TB research has greatly increased ever since^1^. However, we often study the virulence and survival factors of *Mtb* from liquid cultures under “*in vitro* standard conditions”, which usually implies dextrose as carbon source and an *in vitro* or *in vivo* TB model. Anyhow, studies have shown that it is important to control previous conditions of mycobacterial growth before setting up a TB model. For instance, implementation of a detergent-free medium to culture *Mtb* before setting a macrophage infection model, rather than using a medium containing Tween 80, significantly changed the transcriptome and pathways taken by murine macrophages^2^. Likewise, implementation of lipids in *in vitro* dormant models has yielded better mycobacterial recovery and different transcriptomic profiles during *in vitro* resuscitation, compared to dextrose as sole carbon source^3,4^. Lipids are important molecules during the life cycle of *Mtb* as well as during the metabolic dynamics within the host^5,6^. *Mtb* promotes and modulates its phagocytosis using complex lipids of the cell wall^7^ and enters the macrophage through lipid rafts, which mainly contain host cholesterol^8^. Also, *Mtb* recruits the protein TACO allocated in cholesterol-rich zones to avoid the phagosome-lysosome formation^9^. The granuloma formation is also promoted by released lipid microvesicles of *Mtb*, besides other virulence factors^10,11^, which led some authors to suggest the granuloma might be protecting the mycobacteria rather than the host^12^. Nevertheless, *Mtb* is not restricted to its own lipid homeostasis, it also influences host lipids by promoting the recruitment of low density lipoproteins and the formation of foamy macrophages^13^ producing cellular necrosis and caseous granuloma^14^ rich in cholesterol, triglycerides and lactosylceramides^10^. It is proposed that *Mtb* uses mainly lipids as carbon and energy source, rather than dextrose, during latency^3,15,16^. By using ^14^C-labeled lipids, transmission electron microscopy and auramine-nile staining, several studies have demonstrated that fatty acids and cholesterol consumption by *Mtb* lead to cytoplasmic lipid bodies accumulation, cell wall remodelling or synthesis of released lipoproteins^11,16–19^. Moreover, during active TB, *Mtb* evades the host immune cells by exchanging lipids in its cell wall and hiding its earlier identified pathogen-associated molecular patterns (PAMPs)^20^. Also, sputum from TB patients reflects the lipid environment from which mycobacteria is coming from, made up mainly of cholesterol, palmitic, stearic and oleic acids^21,22^. Sputum becomes in turn the microenvironment where the transmissible stage of *Mtb* resides, whose microscopic phenotype and transcriptional profile suggested there is a shift in the lipid metabolism. *Mtb* recovered from sputum showed cytoplasmic lipid bodies revealed with auramine-nile red staining, significant overexpression of the large Kst-R regulon of cholesterol catabolism, overpression of *tgs1* gene required for triacylglycerol synthesis and down-regulation of genes involved in phthiocerol dimycocerosate and phenol glycolipid synthesis indicating cell wall remodeling^23,24^. There is also evidence that hypercholesterolemia increases susceptibility to TB in mice^25^ and a survey in Singapore found positive correlation between a cholesterol diet and an increased risk to active TB in Chinese population^26^. It is thus important to investigate not only the mycobacterial lipid metabolism but also, the mycobacterial response to lipids and the host lipid homeostasis during TB infection. Studies that better mimic the actual metabolic state of *Mtb* during pathogenesis are needed to fully tag therapeutic targets against TB. Although previous studies have focused on the mycobacterial lipid metabolism under different “standard” conditions (drugs, stress), less is known about the transcriptome of *Mtb* in a model that better mimics the lipid environment of the caseous granuloma, rich in cholesterol and fatty acids. This could uncover an unexplored field, potentially useful for the development of new drugs, vaccines and new strategies for controlling TB. In this study we determined the transcriptome of *Mtb* in a lipid-rich dormancy model through RNAseq technology, which has the advantage over qRT-PCR or microarrays, of producing the transcriptome of the organism with a higher resolution, including codifying and non-codifying genes, intergenic regions, small RNAs or antisense transcripts. Moreover, the overexpression of some genes is perfectly quantified without noise, which is usually found in other fluorescence-based systems. This study exposes the transcriptome landscape of *Mtb* H37Rv in the presence of the most abundant lipids inside the host, specifically, cholesterol and long chain fatty acids (C16:0, C18:0, C18:1), using dextrose carbon source as control, under aerobic and hypoxic conditions^27^.

## Results

*In vitro* cultures of *Mtb* were grown in the presence of lipids (cholesterol and fatty acids) as previously described^3^ and parallel cultures with dextrose were used as control. Six conditions were studied: lipid exponential phase (LE), lipid stationary phase (LS), lipid NRP1 stage (LNRP1), dextrose exponential phase (DE), dextrose stationary phase (DS) and dextrose NRP1 (DNRP1). Mycobacterial RNA from all conditions was subjected to RNAseq analysis. Codifying genes (CDS) and non-coding RNAs (ncRNA) were classified according to functional categories listed in Tuberculist^28^, IGRs and sRNA candidates^16,29^, not listed in Tuberculist^28^, were added and featured as genes in the annotation file. After quality control evaluation and trimming of bad.

**Figure 1:**
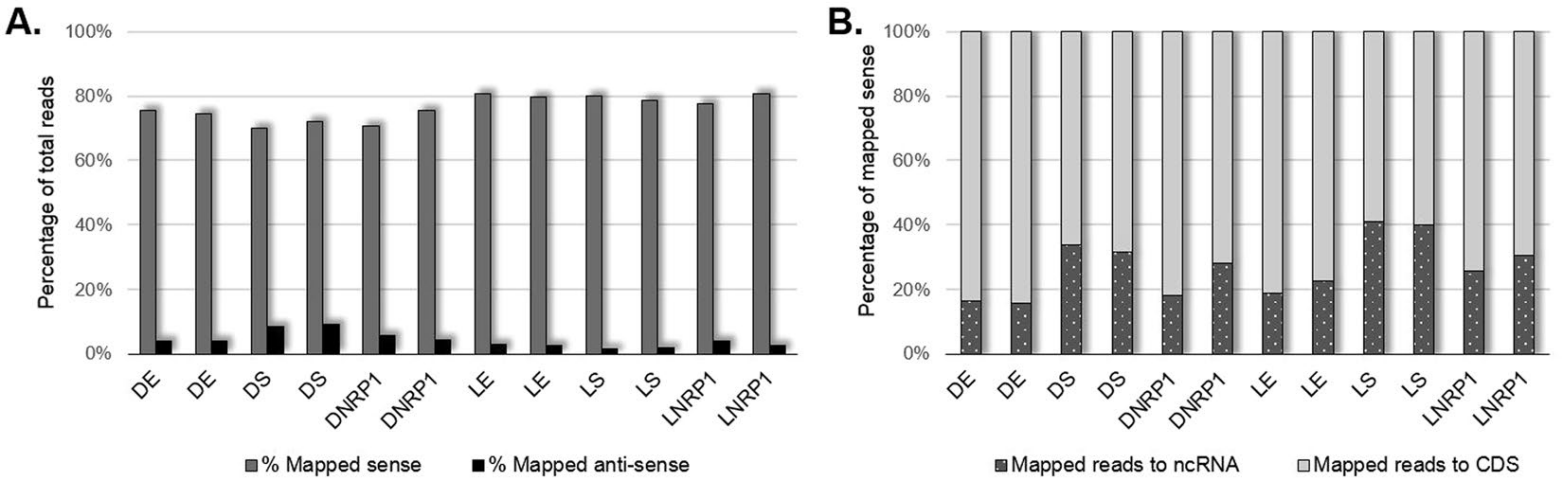
RNA-seq mapping results for every experimental condition and biological replicates. Sense and anti-sense mapping is figured in the chart (**A**). Grey bars show the reads mapped in the sense direction. Not mapped reads were mapped in the anti-sense direction and are represented by black bars. The chart (**B**) show stacked columns of total mapped reads in the sense direction. Gray bars with white dots show the percentage of reads aligned to ncRNA and light gray bars show the percentage of reads aligned to CDS. Detailed data of RNA-seq mapping can be seen in the Supplemental Material, Table S3.

**Figure 2:**
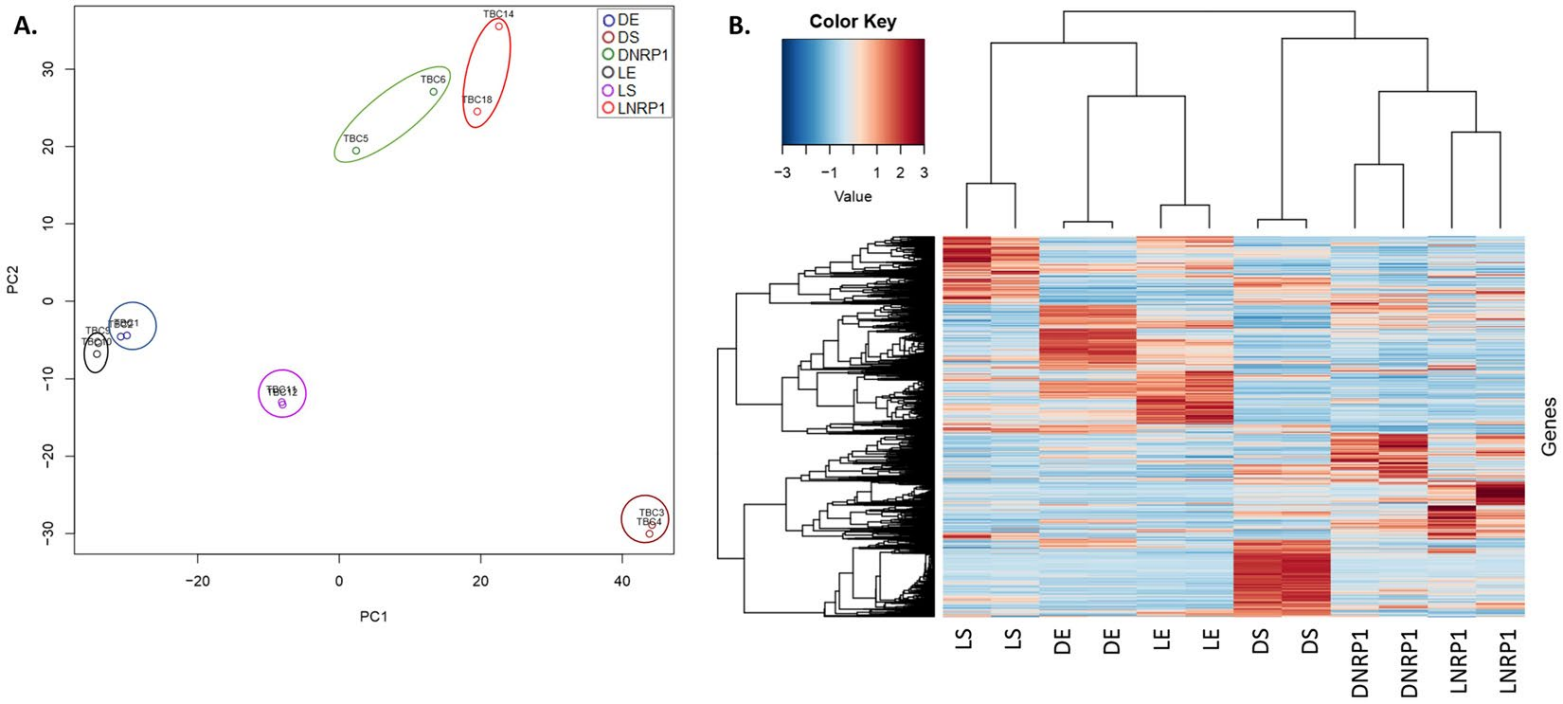
Plots of the principal component analysis (**A**) and the clustering-heatmap (**B**) of all genes after normalization using edge R. For the PCA plot, the data was rlog transformed. For the clustering heatmap, normalized counts were rescaled between −3 and 3, clustering was based on Pearson correlation. The six experimental conditions clustered with their biological replicates.

Qualitative reads, a mean of 54.8 ± 9.4 million paired reads per individual library were detected. 76.3 ± 3.9% of total reads mapped in the sense direction, while 4.3 ± 2.5% mapped in the anti-sense direction. It is worthy of notice that DS samples had slightly more anti-sense reads (9 ± 0.6%) than the rest of the conditions (Fig. 1A). Deep sequencing analysis of the sense reads showed that more ncRNAs were found in DS, LS and LNRP1, 32 ± 1.4%, 41 ± 0.8% and 28 ± 3.3% of total aligned sense-reads, respectively (Fig. 1B). Finally, the principal component analysis (PCA) and the clustering heatmap plots showed that the samples clustered by their biological replicates (Fig. 2).

### Differential gene expression analysis for lipid conditions

**Figure 3:**
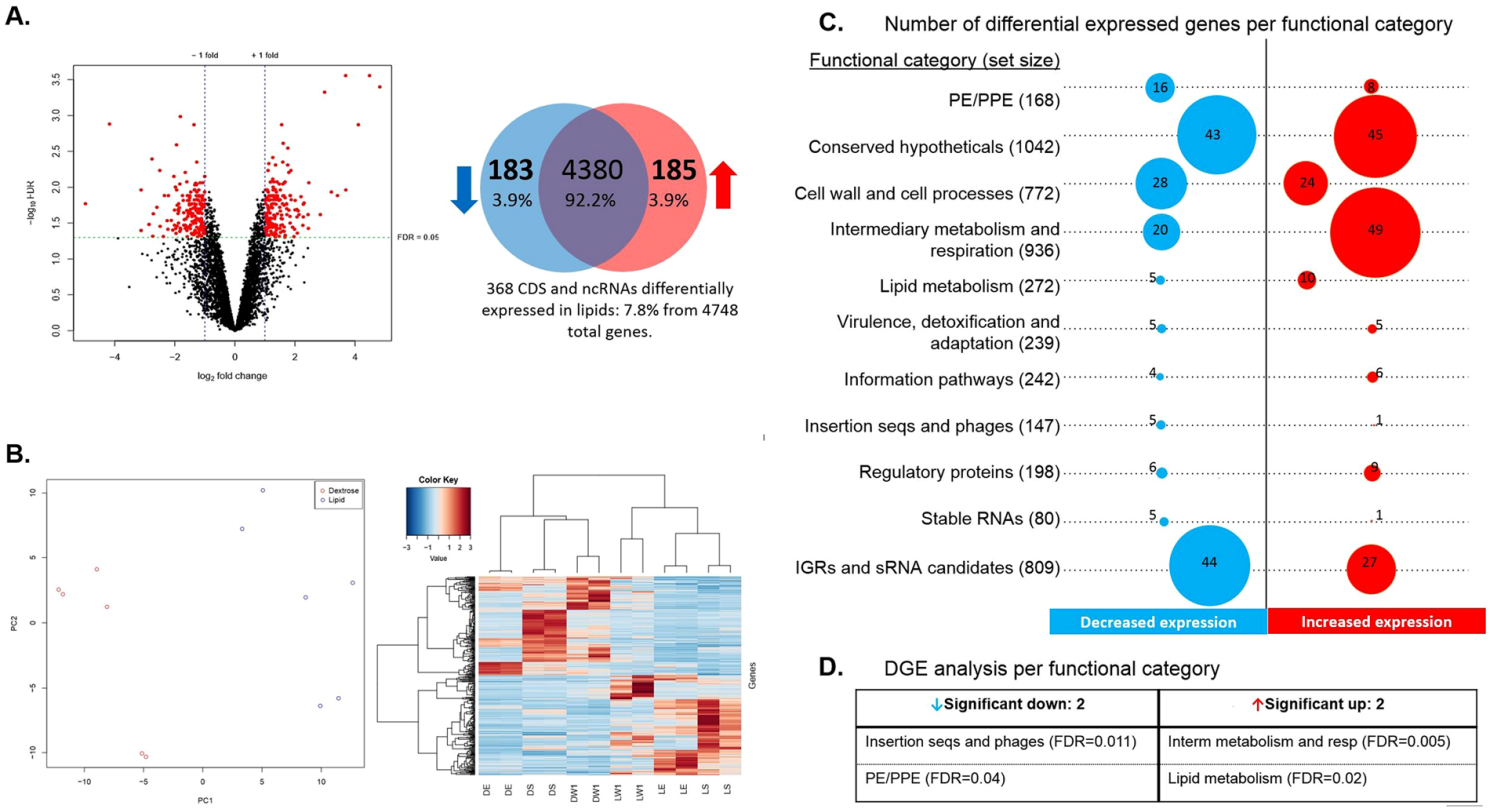
Differential gene expression of *Mtb* H37Rv in presence of lipids. Conditions with dextrose were used as control and growth stages were used as batch effect. Counts were normalized using edge R, all genes with a CPM greater than 1 in at least 2 samples were used in the analysis. 368 significant differentially expressed genes were found. (**A**) Volcano plot (on the left) and its Venn diagram (on the right) where 185 genes with diminished expression and 183 genes with increased expression in presence of lipids are specified. (**B**) PCA and heatmap plots of the 368 differential expressed genes that were rescaled between −3 and 3, clustering was based on the Pearson correlation. (**C**) Number of differentially expressed genes by functional categories are shown, circle sizes are proportional to the number of genes with significant differential expression, total number of genes per functional category listed in tuberculist are indicated in parenthesis. (**D**) Differential functional categories analysis. Only significant expressed categories (FDR < 0.05) are indicated.

To investigate the impact of lipids during the different stages, differential gene expression (DGE) analysis for the first general lineal model was performed comparing lipid conditions vs dextrose (D vs L). Six replicates for each dextrose and lipid group were performed, ordered in three subgroups (exponential, stationary and NRP1). Differentially expressed genes were filtered using a false discovery rate (FDR) of <0.05 and a log_2_ fold change >1 or <−1 for biological significance. After filtering, 368 genes were found differentially expressed in the presence of lipids (Fig. 3 and Table S1 in the Supplemental Material) representing 7.8% of the total covered genes. From these, 183 genes decreased and 185 increased their expression (Fig. 3A). As shown in the PCA plot and in the heatmap visualization (Fig. 3B), the 368 genes clustered by the carbon source (lipid or dextrose) and by biological replicates. Regarding the functional categories, most of the differentially expressed genes belonged to the categories of conserved hypothetical proteins, cell wall and cell processes, intermediary metabolism and respiration and the appended category of IGR and sRNA candidates (Fig. 3C). These results were not unexpected given the substantial number of genes that are included in these four categories compared with the others in the whole genome of *Mtb*. For this reason, we proceeded to perform a differential functional category analysis, which took into account the category size. Results showed that the category of insertion sequences and phages as well as the PE/PPE category decreased their expression and, as expected, the presence of lipids prompted the expression of the lipid metabolism category, together with the intermediary metabolism and respiration category (Fig. 3D and Supplemental Material Table S4). Specifically, the ten genes with increased expression from the lipid metabolism were *ltp2*, *pks2*, *tgs2*, *mutA*, *rv3087*, *rv1425*, *fadA5*, *fadD2*, *fadE7* and *fadE9*. Whilst the genes with diminished expression from the insertion sequences and phages category were *rv2646*, *rv3427c*, *rv2647*, *rv1199c* and *rv2512c* and from the PE/PPE family were *PE_PGRS62*, *PE15*, *PE21*, *PE22*, *PE23*, *PE25*, *PE3*, *PE8*, *PPE1*, *PPE21*, *PPE22*, *PPE23*, *PPE24*, *PPE34*, *PPE57* and *PPE67*. It also draws our attention the *MTS2823* ncRNA from the category of stable RNA, since it was the only one from this category with significant and differential increased expression in the presence of lipids, in the first general linear model (D vs L).

Additionally, a differential pathway analysis (DPA) was performed using the KEGG database^30^, to determine the impact of lipids over all mycobacterial phases. Again, dextrose was used as control, growth phases were set as a batch effect. This analysis predicted significantly induced or repressed pathways, either in a direct or indirect mode. The results of this analysis confirmed the induction of the steroid degradation and fatty acid degradation pathways (see Supplementary Material Table S5). Additionally, lipids promoted two-component systems, pyruvate metabolism, carbon metabolism, lysine degradation, valine, leucine and isoleucine degradation, tryptophan metabolism, butanoate metabolism and oxidative phosphorylation.

### Differential gene expression analysis for every stage of growth of Mtb

**Figure 4:**
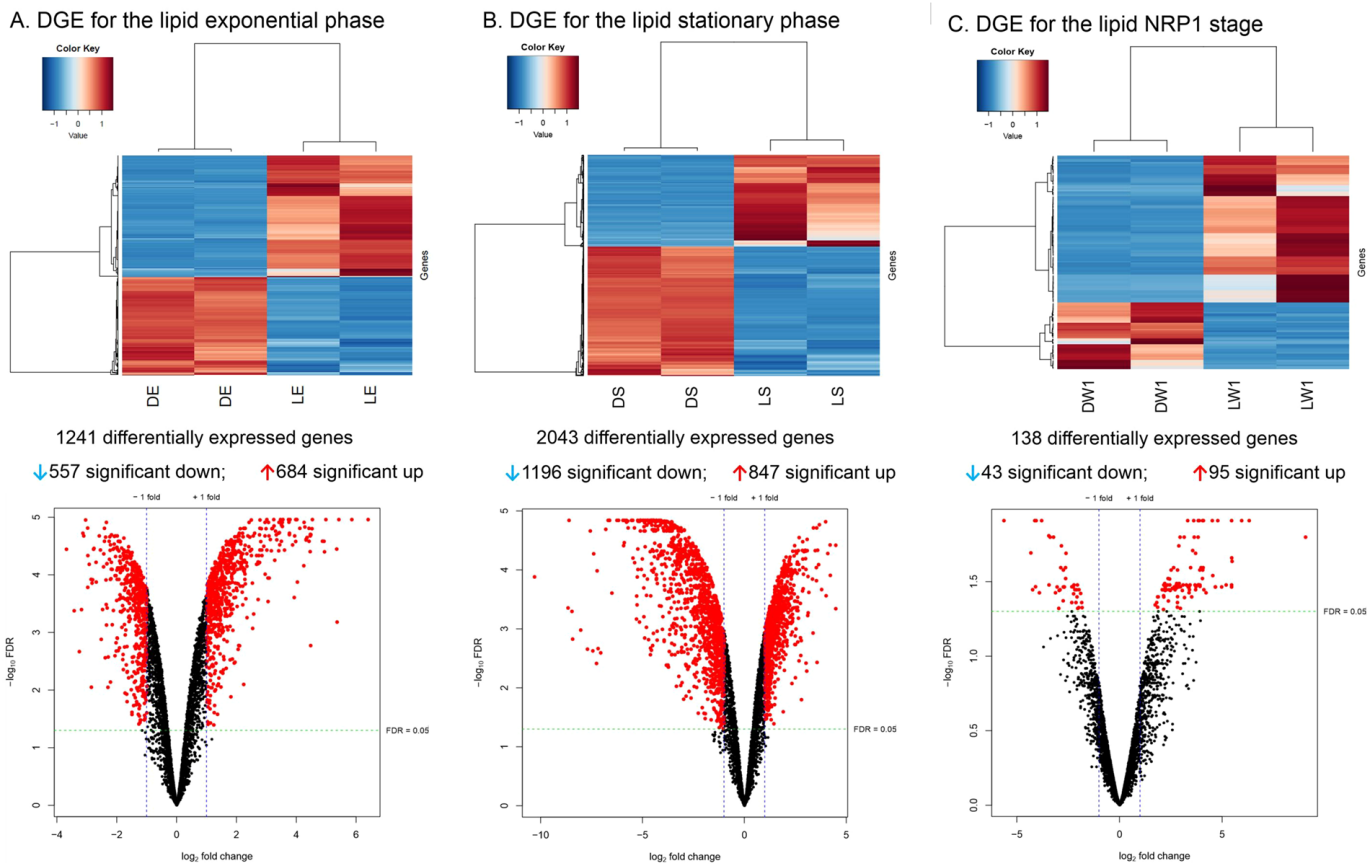
Differential gene expression of *Mtb* H37Rv in every stage of growth in presence of lipids. Dextrose conditions were used as controls. The heatmap and volcano plots are shown in figure (**A**) for the 1241 differentially expressed genes in LE, figure (**B**) shows the 2043 differentially expressed genes in LS and figure (**C**) shows the 138 differentially expressed genes in LNRP1. Normalized counts in the heatmap were rescaled between −3 and 3 with clustering based on Pearson correlation and differentially expressed genes were considered significant using a false discovery rate of <0.05 and a log_2_ fold change >1 or <−1.

**Figure 5:**
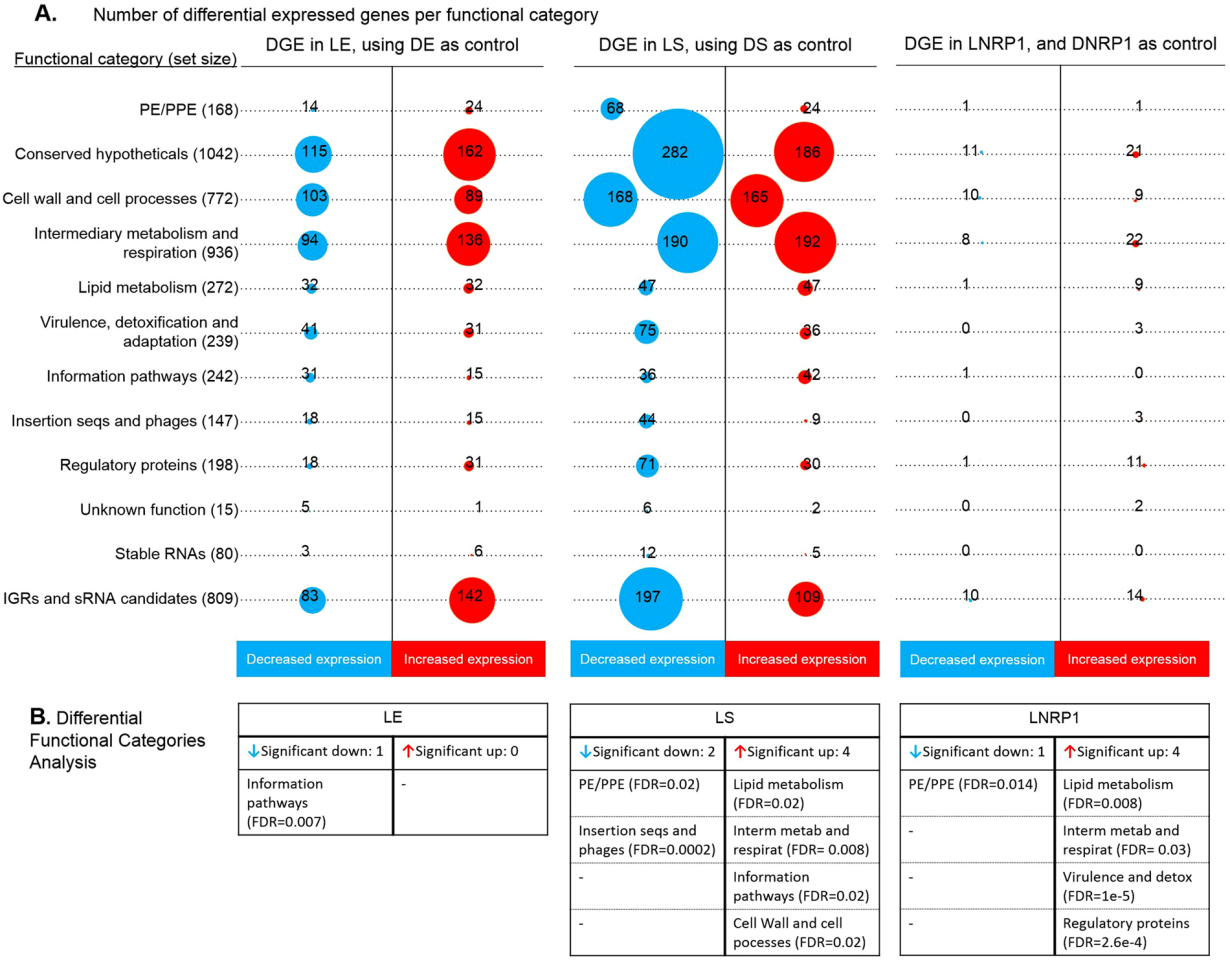
Differential gene expression per functional categories of every stage of growth of *Mtb*. (**A**) The number of differentially expressed genes are shown by functional categories, where circle sizes are proportional to the number of genes with significant differential expression. Blue circles are used for genes with diminished expression and red circles are used for genes with increased expression. Total number of genes per functional category listed in tuberculist are indicated in parenthesis. (**B**) Differential functional Categories analysis are indicated in tables for every stage of growth, wherein the mean log_2_ fold change is significantly different from the general mean log_2_ fold change at a FDR <0.05.

Three linear models of DGE were made for the lipid growth stages LE, LS and LNRP1, with dextrose cultures (DE, DS and DNRP1) as controls. This analysis identified 1241 (557 down- and 684 up-regulated) differentially expressed genes in LE; 2043 (1196 down- and 847 up-regulated) differentially expressed genes in LS, while the LNRP1 stage showed the smallest number of genes, with 138 (43 down- and 95 up-regulated) differentially expressed (Fig. 4 and Supplementary Material Table S2). The number of genes differentially expressed per category can be seen in Fig. 5A. Again, the biggest categories: conserved hypothetical proteins, cell wall and cell processes, intermediary metabolism and respiration and the appended IGRs and sRNA candidates, had the higher number of significantly expressed genes. The differential functional categories analysis took into account the size of every category (Fig. 5B and Supplemental Material Tables S6, S7 and S8). Through this analysis, it was possible to define the categories up- and down-regulated in every phase of growth. The PE/PPE category was significantly down-regulated in the two models of dormancy in the presence of lipids (LS and LNRP1), while the lipid and the intermediary metabolism and respiration were significantly up-regulated in both stages. Among the differences between these two stages, we had two categories up-regulated in LS, information pathways and cell wall and cell processes categories, while the virulence and regulatory proteins categories were up-regulated during the lipid hypoxia LNRP1. On the other hand, the exponential stage only had the information pathways category significantly down-regulated.

### The main core lipid response

**Figure 6:**
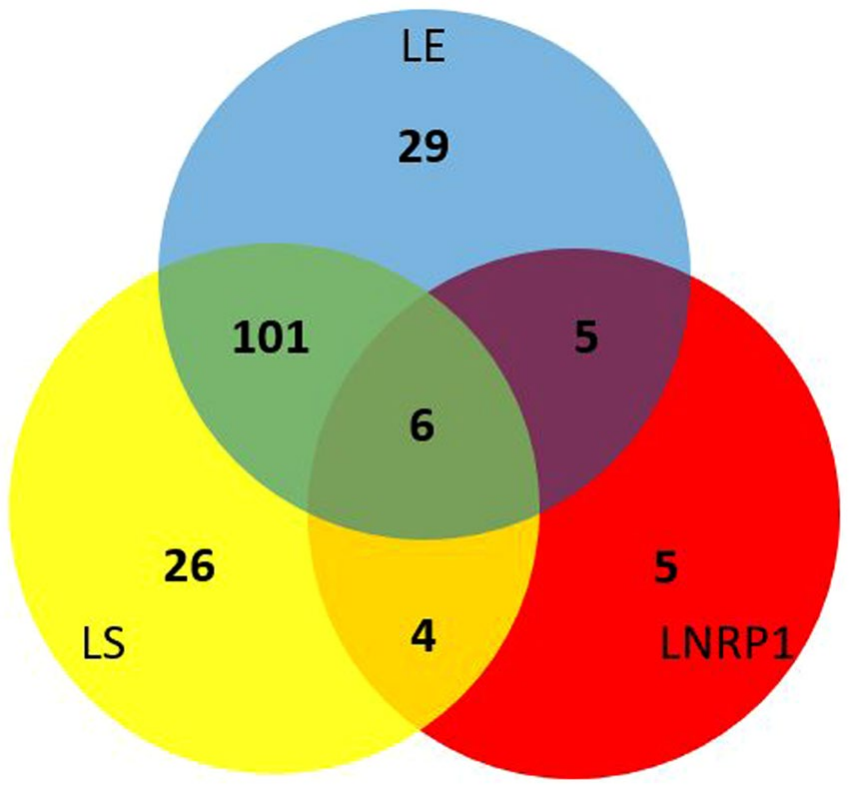
Core of genes induced by the presence of lipids.

Figure 6 shows the number of genes that belong to the core induced by the presence of lipids. It shows a Venn diagram with the set of significantly increased genes in lipids, from the first DGE analysis (D vs L), that overlaped with significantly increased genes in the subsequent DGE lineal models per stage of growth ([DE vs LE], [DS vs LS] and [DNRP1 vs LNRP1]). Although all of them were significantly overprexpressed in the presence of lipids, only six genes from the comparison D vs L overlapped in the three stages of *Mtb*, and they were called *“the main core lipid response”*, comprising *Rv3161c*, *Rv3160c*, *Rv0678*, *Rv1217c*, *PPE53* and *che1*. Genes from the remain sets and detailed gene expression data can be checked in the Supplementary Material, Table S1 and S2.

### qRT-PCR validation

**Figure 7:**
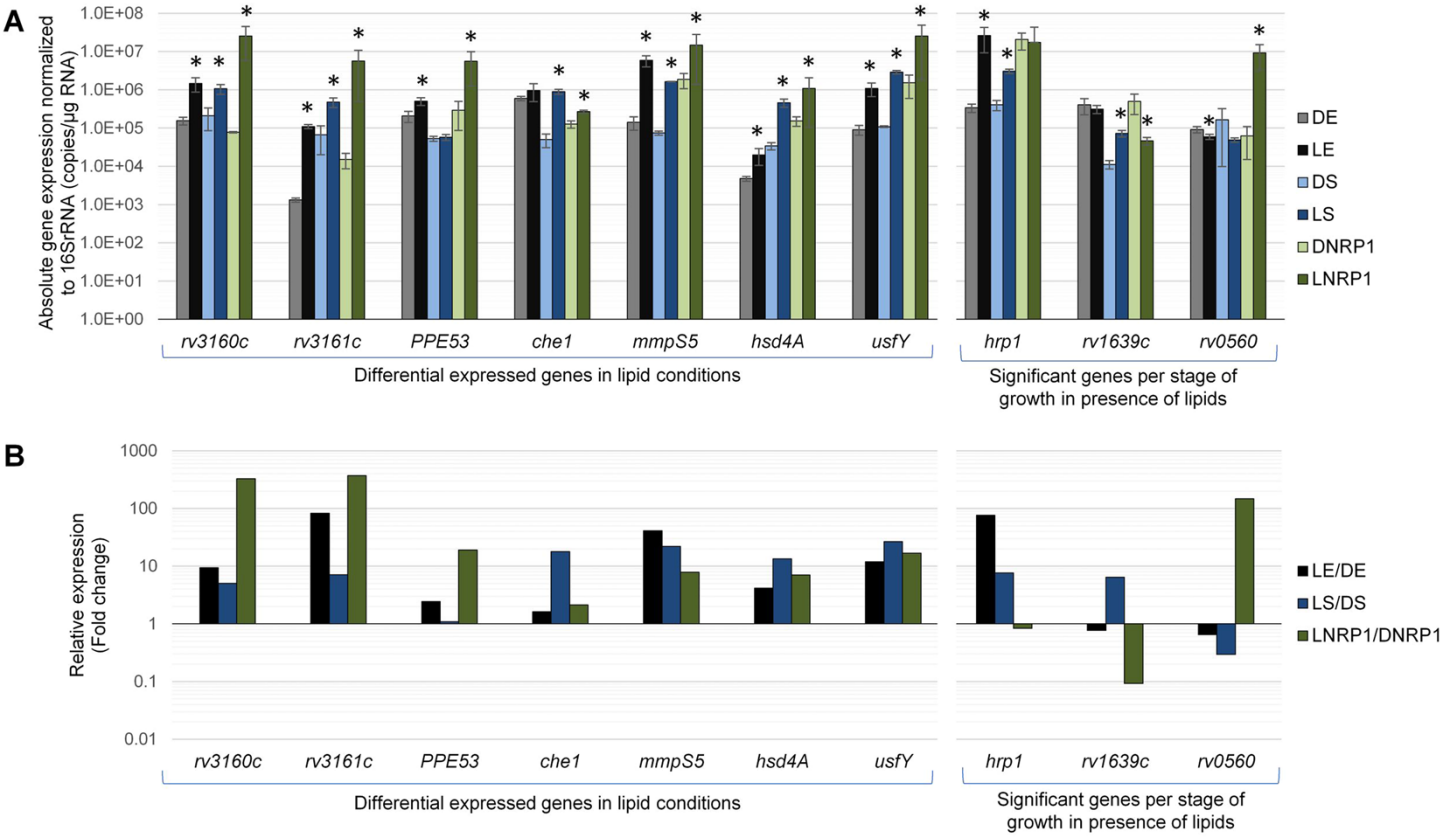
Validation of ten differential expressed genes in lipid conditions by qRT-PCR. (**A**) Absolute gene expression during DE (gray bars), LE (black bars), DS (light blue bars), LS (dark blue bars), DNRP1 (light green bars) and LNRP1 (dark green bars) conditions. Data were normalized to the number of copies of *rrs* gene and results are expressed as log_10_ of the mean ± SD number of copies per microgram of RNA. Asterisks indicate significant diference between a given lipid stage compared to its respective dextrose stage (LE vs DE, LS vs DS and LNRP1 vs DNRP1), with *P* < 0.05 considered statistically significant. (**B**) The relative quantification is expressed as the ratio of [transcription in LE/transcripion in DE] (black bars), the ratio of [transcription in LS/transcription in DS] (blue bars) and the ratio of [transcription in LNRP1/transcription in DNRP1] (green bars).

Validation of RNAseq results was made through qRT-PCR, for which a set of ten significantly up-regulated genes in the presence of lipids were confirmed. Four technical replicates for each two biological replicates were tested for gene expression at every stage of growth in lipids or dextrose. Seven of the most significantly upregulated genes from the first linear DGE analysis (comparing L vs D) were chosen: *rv3160c*, *rv3161c*, *PPE53*, *che1*, *mmpS5*, *hsd4A* and *usfY*. Additionally, three significantly upregulated genes from the DGE analysis for every stage of growth were chosen based on lipid phase exclusivity: *hrp1* was overexpressed in LE, *rv1639c* in LS and *rv0560* in LNRP1. Absolute gene expression of the ten genes was determined and normalized to 16 S rRNA gene (Fig. 7A). For relative quantification, the absolute transcription level of genes in every lipid stage was compared with their respective dextrose control (LE/DE, LS/DS and LNRP1/DNRP1; Fig. 7B).

Similar to the DGE results, the seven chosen genes from the comparison L vs D were up-regulated in the three lipid conditions (LE, LS and LNRP1) by qRT-PCR, though the upregulation of *PPE53* in LS and *che1* in LE were not statistically significant. Likewise, *hrp1*, *rv1639c* and *rv0560* were the most expressed in LE, LS and LNRP1, respectively, although *hrp1* was not exclusive of LE due to the given significant overexpression in LS as well (Fig. 7 and Supplemental Material S9 for statistics).

## Discussion

Previous studies have shown a close link between persistent mycobacteria and drug tolerance^31^. Persistent mycobacteria are presumed to be the reason for many treatment failures, even in the presence of drug susceptible mycobacteria. The problem can reside in multiple mycobacterial phenotypes, including the persistent non-culturable mycobacteria, which cannot be subjected to DST. All these populations have in common the presence of host lipids, at several levels, from host membranes to caseous granuloma or sputum. In fact, *Mtb* not only modulates its own lipid metabolism but also influences the host lipid metabolism to be fully surrounded by lipids at one point of its life cycle^5^. Through RNAseq we confirmed that lipid accession is one more stimulus, besides other stress factors, by which *Mtb* increases their persistent population. The differences between the Rodriguez *et al*., study^16^ and ours are mainly based on the culture conditions and the bioinformatic analysis. Rodriguez *et al*. selected the even-length long-chain fatty acids (LC-FAs) as the sole carbon source. In the present study, to gain more insight into the *M. tuberculosis* adaptation to a fatty acid environment, we implemented cholesterol together with the fatty acids as carbon source, considering the propionyl-CoA pool, which is a central metabolite to produce methyl-branched lipids of the cell wall. Another difference is the hypoxic condition evaluated in the presence of lipids and dextrose of this study. Limited oxygen is a feature of granulomas and hypoxic models have been frequently used to study dormant mycobacteria. Additionally, the library construction, normalization of the data, DGE analysis and statistical analysis were completely different between both studies. Yet, the study of Rodríguez *et al*., must be considered since the authors confirmed that fatty acids prompted a phenotype of dormancy and the study highlighted relevant features of the transcriptome of *Mtb* during fatty acid consumption.

**Figure 8:**
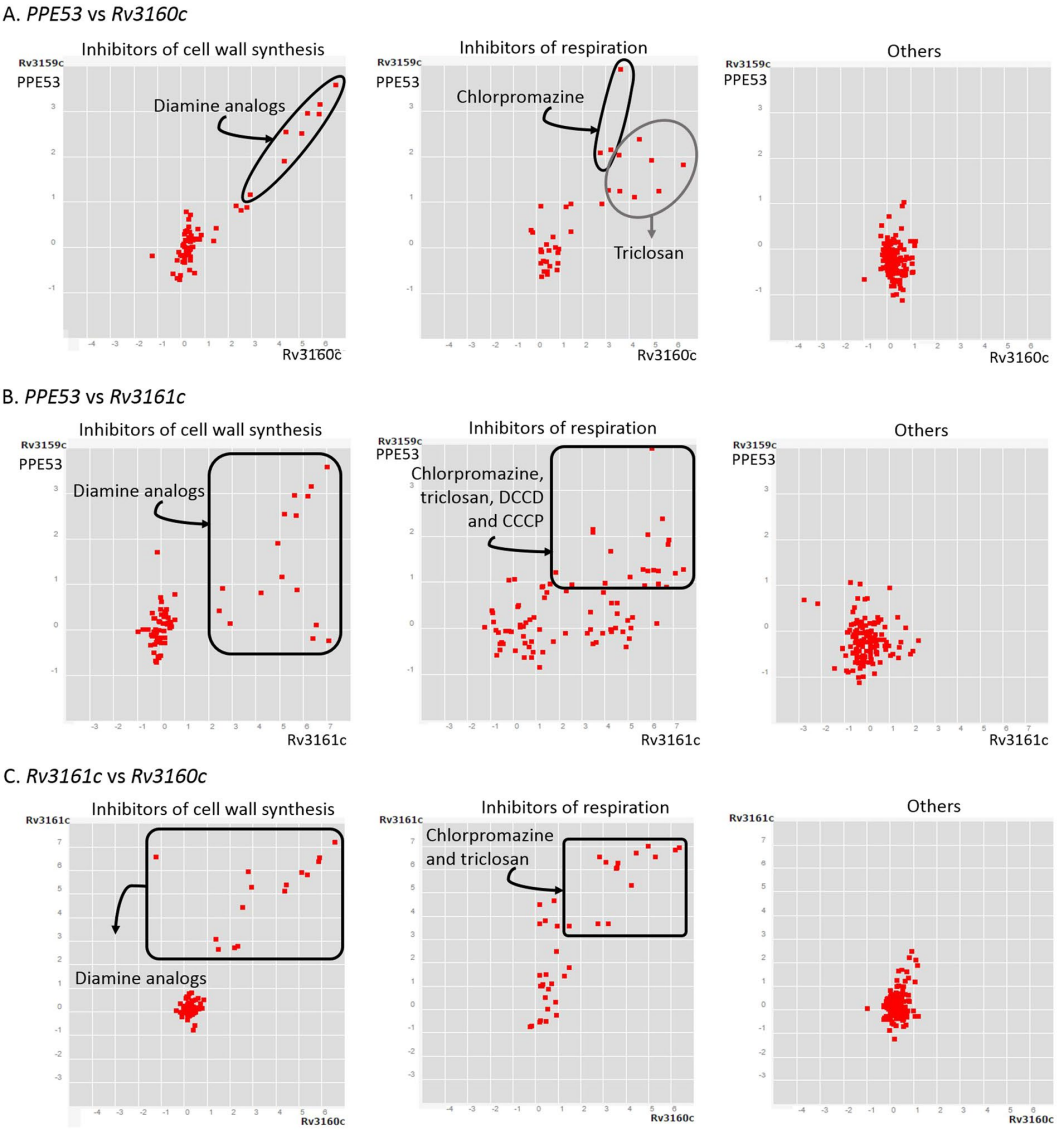
Correlated gene expression among PPE53, Rv3160c and Rv3161c. Plots show data reported by Boshoff and colleagues through microarray assay, which is deposited in the correlation catalog of the TBDB data base^41,42^. (**A**). Comparison between PPE53 and Rv3160c; (**B**). Comparison between PPE53 and Rv3161C; (**C**). Comparison between Rv3161c and Rv3161c. Black frames and circles highlight positive correlation between two genes in samples of *Mycobacterium tuberculosis* under diferent conditions. Gene expression responses of Mtb to 76 treatment groups are represseented by red dots. Plots resumed experimental categories: 1) samples of Mtb under inhibitors of cell wall synthesis (left), 2) samples of Mtb under diferent inhibitors of respiration (middle) and 3) “others” (on the right) inlcludes 8 categories more, such as acidified médium, agents that affect DNA integrty or topology, aromatic amides that can be hydrolyzed intracellylary, growth associated with dosR regulon, inhibitors of protein synthesis, minimal medium with palmitate or succinate as carbon, starvation and transcriptional inhibitors. It can be seen that PPE53, Rv3160c and Rv3161c are mainly co-expressed in compounds that affect cell wall synthesis and respiration. On the other hand, these genes are not induced by compounds from the cathegory “others”.

**Figure 9:**
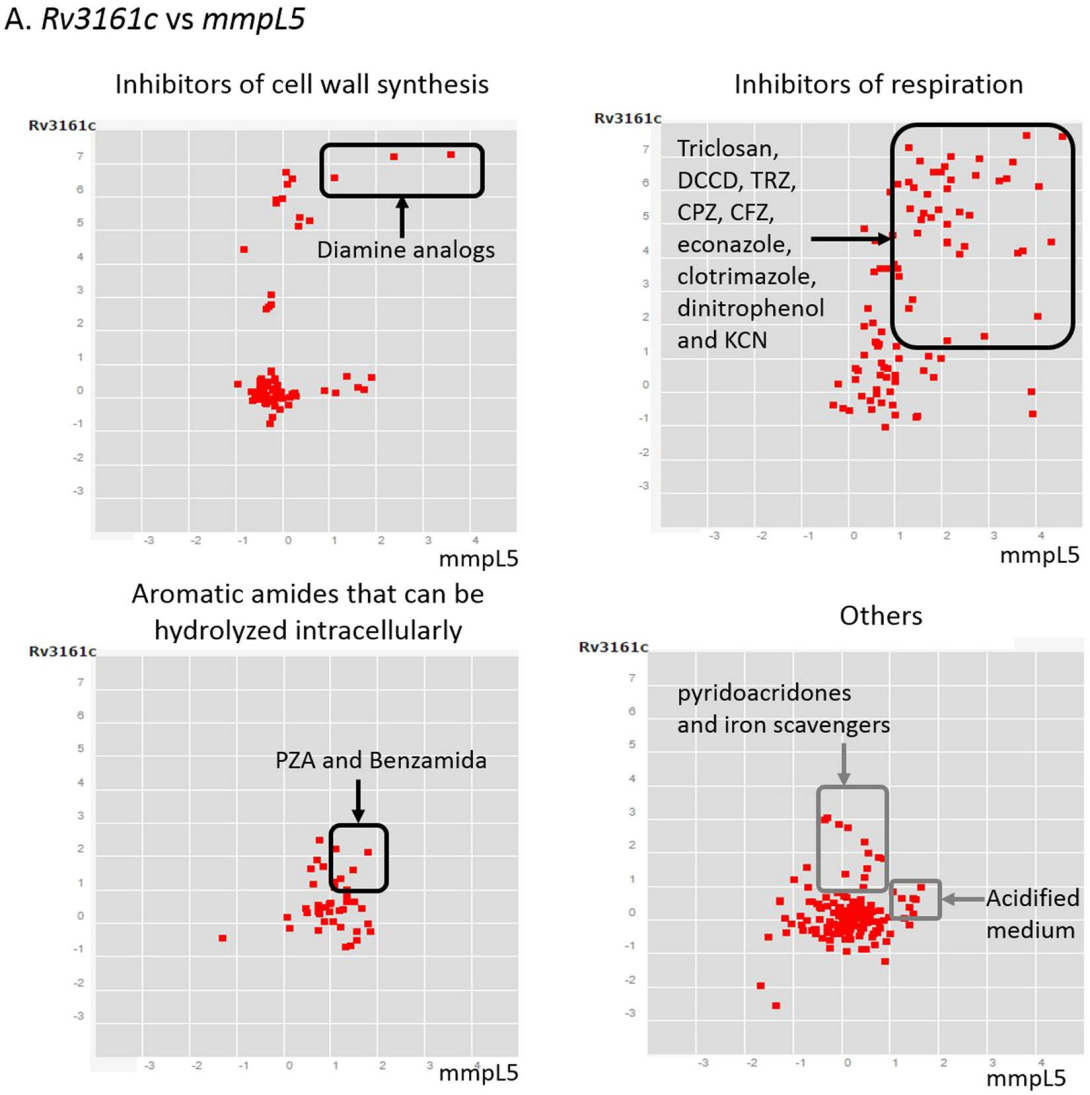
Correlated gene expression between *Rv3161c* and *mmpL5*. Plots show data reported by Boshoff and colleagues^33^ through microarray assay, which is deposited in the correlation catalog of the TBDB data base^41,53^. Black frames highlight positive correlation between *Rv3161c* and *mmpL5* (>1 for both) in samples of *Mycobacterium tuberculosis* under diferent conditions. Gene expression responses of *Mtb* to 76 treatment groups are represseented by red dots. Plots resumed experimental categories: 1) samples of *Mtb* under inhibitors of cell wall synthesis (upper left), 2) samples of *Mtb* under diferent inhibitors of respiration (upper right), 3) in presence of aromatic amides (lower left) and 4) “others” (lower right) inlcludes 7 categories more, such as acidified médium, agents that affect DNA integrty or topology, growth associated with dosR regulon, inhibitors of protein synthesis, minimal medium with palmitate or succinate as carbon, starvation and transcriptional inhibitors. DCCD: Dicyclohexylcarbodiimide, TRZ: Thioridazine, CPZ: Chlorpromazine, CFZ: Clofazimine, KCN: Potassium cyanide, PZA: Pyrazinamide^33,54^. It can be seen that *Rv3161c* and *mmpL5* are mainly co-expressed in compounds that affect cell wall synthesis and respiration as well as in presence of aromatic amides. Besides, these genes are not co-expressed by compounds from the cathegory “others”.

Here, *Mtb* differentially expressed a core of 368 genes in the presence of lipids (183 down- and 185 up-regulated). Our study included lipids in aerobic exponential growth, aerobic stationary phase and hypoxic stage (NRP1), the last two better mimicking dormancy^27,32^. Since lipids are present and involved in most stages of *Mtb* during TB pathogenesis^6^, these 368 genes may be clues in the mycobacterial metabolism during its survival. The genes *Rv3161c*, *Rv3160c*, *Rv0678*, *Rv1217c*, *PPE53* and *che1*, form what we call *“the main core lipid response”*; and have been linked to processes of drug detoxification, iron acquisition and efflux and transport systems also co-expressed with the *mmpL5*-*mmpS5* operon. The correlated overexpression of *Rv3160c-*RV3161c and *PPE53* have also been found in the presence of cell wall and respiration inhibitors (Fig. 8)^33,34^. Thus, it is proposed that this operon is required for detoxification of drugs^35^, although there is no evidence of its direct involvement in drug resistance^36^. This operon is co-expressed with *mmpS5-mmpL5* in the presence of drugs (Fig. 9)^33^ as it happened in the presence of lipids of our study. The MmpS5-mmpL5 efflux pump is not only related to drug resistance but siderophore transportation^37,38^. Another gene from the mean core lipid response related to iron utilization was *che1*, whose codified product is a ferrochelatase. This gene resides in a sulfur reduction operon, constituted by *sirA*, *cysH*, *che1*, *ggtB* ^39,40^. The role of Che1 is to insert iron into a siroheme.

*Rv1217c* (from the mean core lipid response) is predicted to be part of the operon integrated by *Rv1216c*, *Rv1217c*, *Rv1218c* and *Rv1219c* ^39^. All were differentially overexpressed in the presence of lipids in our study (Table S1). Likewise, this operon is co-expressed with *mmpS5*-*mmpL5* in the presence of the drugs^41^ as it happened in the presence of lipids of our study.

It is also important to mention the overexpression of an efflux pump codified by *mmr* gene and a sulfur reduction operon (*Rv2391* – *Rv2394*). All these genes are linked somehow and were significantly overexpressed in the presence of lipids in this study (Table S1). Mmr is an efflux pump involved in transport of multidrugs and codified next to Rv3066 that encodes a transcriptional regulator belonging to the deoR-family. They belong to the same operon^39^ and *Rv3066* is predicted to repress *mmr* ^42^. Rv3066 was not up-regulated under lipid conditions; in fact, its transcription was significantly diminished in LS stage allowing transcription of *mmr*. Hence, we hypothesize that all genes mentioned above are coordinated to act not only at the level of intracellular redox balance but also preparing the machinery that confers drug tolerance to *Mtb*.

According to the differential functional category analysis (DFCA), it is not surprising that lipids mostly induced the expression of the lipid metabolism category (Fig. 3D). This was also confirmed by a differential pathway analysis using KEGG^30^, which showed activation of fatty acid degradation and steroid degradation pathways. Specifically, essential genes for *in vitro* growth in cholesterol^43^ such as *cyp125*, *kstD*, *hsaB*, *hsaC*, *fadA5*, *ltp2* and *pks2* were induced, as well as genes for lipid degradation, including *fadE9*, *fadD2*, *fadE7*. Additionally, there were genes involved in lipid biosynthesis, such as for triacylglycerol (TAG) (*tgs2*, *Rv3087* and *Rv1425*) and *mutA*, necessary for multimethyl-branched lipid biosynthesis through the methylmalonyl pathway. Based on this, we can assume that *Mtb* used cholesterol and fatty acids as carbon source and synthetized new macro-lipids, either for lipid deposition as TAG or for cell wall remodeling.

There were eight PE/PPE genes overexpressed in the DGE core (*PPE53*, *PE_PGRS54*, *PE_PGRS5*, *PE_PGRS10*, *PE_PGRS57*, *PE_PGRS15*, *PE_PGRS53*, *PE_PGRS33*). From these, only *PPE53* was strongly up-regulated in the three lipid phases of this study. The role of PE/PPE proteins remains uncertain but evidence suggests that they confer virulence variability to mycobacteria. A system of oxidative stress was significantly overexpressed in the lipid stationary phase (LS) compared to cultures in dextrose (DS). This system includes the enzymes KatG, SodA and AhpC^44^, all essential for virulence^44^. Also, during stationary phase, lipids induced expression of *vapC1*, *vapC22*, *vapB1*, *vapB10*, *vapB48* and *vapB46*, while during exponential phase (LE) and hypoxia (LNRP1), there was overexpression of complete modules vapB22/vapC22 and vapB9/vapC9, respectively (Table S2), suggesting that lipids promote a higher survival through vapBC components in exponential and hypoxic phases and may contribute to persistence population appearance.

Lipids also induced the overexpression of non-codifying genes in this study. At least one third of the *Mtb* transcriptome, including rRNA, belongs to non-codifying RNAs (ncRNAs)^45^. Our results confirmed that the transcriptome of *Mtb* drifts towards a greater proportion of non-codifying transcripts, out of rRNA, under different *in vitro* stress conditions as previously reported^16,45,46^. In this study, those ncRNA transcripts were more abundant in the stationary phases and under lipid hypoxia (Fig. 1B; DS, LS and LNRP1), confirming previous studies that suggest that ncRNA are strongly involved in mycobacterial survival. The small ncRNA *MTS2823* was the only significantly overexpressed ncRNA found from the stable RNA’s category (Fig. 3C). The DGE analysis showed that up-regulation of *MTS2823* was co-expressed with sigE which was up-regulated in the lipid exponential phase with significant down regulation of *prpC*/*prpD* genes, needed for the methylcitrate cycle (Table S2). There was also overexpression of *desA3* in the lipid hypoxic condition (LNRP1).

**Figure 10:**
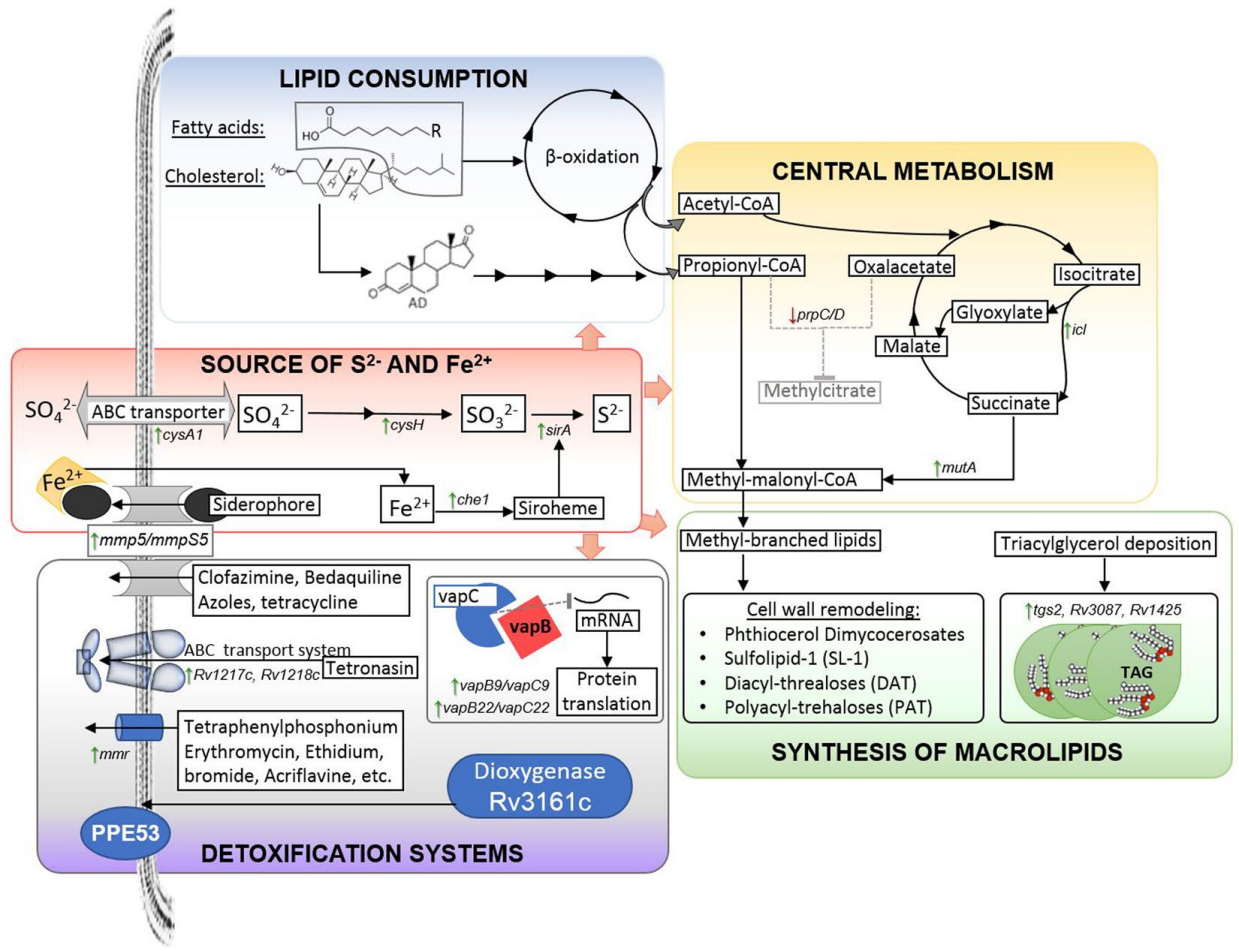
Principal metabolic pathways activated by the core lipid response.

In summary, using RNAseq we determined the transcriptomic profile induced by a mixture of cholesterol, palmitic, stearic and oleic acids, in *Mtb* H37Rv, over three metabolic stages, exponential and stationary stages, as well as for the non-replicating persistence stage 1 of hypoxia (NRP1). The transcriptomic profile suggested that cholesterol and fatty acids were used as carbon source and induced what we have called the mean core lipid response. This mainly induced a machinery that confers drug tolerance to a vast variety of drugs but also ensures the maintenance of iron caption and sulfide production, necessary for enzymes and coenzymes needed for redox balance and production of central metabolites, such as acetyl-CoA and methyl-malonyl-CoA, needed for *de novo* lipid biosynthesis (Fig. 10). As additional studies confirm the importance of lipids during the TB pathology, the core lipid response may give us some clues to target relevant pathways in the *Mtb* metabolism and find better therapeutic targets, for antibiotic and vaccine development as well as for better therapeutic regimens of TB patients.

## Methods

### Bacterial strain and growth conditions

*Mtb* cultures were handled under the specific biosafety recommendations established by WHO^47^. *Mtb* H37Rv was cultured in Dubos broth (Difco), without glycerol, containing 0.5% albumin (Sigma Fraction V), supplemented with either 0.2% dextrose or a lipid mixture (oleic acid, palmitic acid, stearic acid, at final concentration of 0.001% each, plus 0.01% cholesterol). Three growth phases of *Mtb* were cultivated for each carbon source (dextrose or lipids), as it was previously described by Soto-Ramirez *et al*., 2017. Specifically, *Mtb* was grown until reaching 1) its aerobic exponential phase (DE: dextrose exponential phase and LE: lipid exponential phase), 2) its aerobic stationary phase (DS: dextrose stationary phase and LS: lipid stationary phase) and 3) its NRP1 stage of dormancy (DNRP1: dextrose NRP1 stage and LNRP1: lipid NRP1 stage), for which, a parallel culture supplemented with methylene-blue (1.5 mg mL) was used as an indicator of oxygen depletion. A total of six experimental conditions were assessed and at least two biological replicates were done for all conditions. For lipid stages *Mtb* was grown to exponential stage containing 2.4 × 10^6^ ± 9 × 10^5^ CFU/mL, OD_600_ between 0.3 to 0.5, at day 7 of incubation, stationary stage containing 2.9 × 10^6^ ± 2 × 10^6^ CFU/mL, OD_600_ between 0.7 to 0.9, at day 15 of incubation and NRP1 stage containing 2.7 × 10^6^ ± 8 × 10^5^ CFU/mL, OD_600_ between 0.3 to 0.4, at day 8 of hypoxia. For dextrose stages *Mtb* was grown to exponential stage containing 8.3 × 10^6^ ± 1.86 × 10^6^ CFU/mL, OD_600_ = 0.3, at day 7 of incubation, stationary stage containing 2.9 × 10^8^ ± 9.43 × 10^6^ CFU/mL, OD_600_ = 0.6, at day 15 of incubation and NRP1 stage containing 5.3 × 10^6^ ± 1.1 × 10^6^ CFU/mL, OD_600_ = 0.3, at day 5 of hypoxia.

### RNA isolation

Total RNA was isolated from all *Mtb* cultures as previously described^48^. All work involving viable *Mtb* was performed in a dedicated Biosafety Level 3 (BSL 3) laboratory. In short, each culture (200 mL) was harvested by centrifugation and pellets were resuspended in 2 mL of lysis buffer (6 M guanidinium chloride, 0.1% Tween 80, 1 mM 2-mercaptoethanol, 10 mM EDTA). Cells were lysed mechanically in a FastPrep cell disrupting equipment (Thermo Scientific) with 150 μm–to 200 μm glass beads (Sigma-Aldrich) by performing three lysis cycles of 45 s, at 4.0 m/s followed by two lysis cycles of 15 s at 6.5 m/s. Nucleic acids were extracted with phenol-chloroform-isoamyl alcohol (25:24:1) and RNA was precipitated with 0.4 volume of absolute ethanol. Total RNA was purified three times with Trizol reagent (Invitrogen), RNA integrity was analyzed with a Bioanalyzer (Agilent Technologies), quantified by spectrophotometry with the NanoDrop ND-1000 (Thermo Scientific) and DNA absence was evaluated by qPCR, using primers for amplification of the gene *rpsL* (see Supplementary Material, Table S10). Finally, RNA was dissolved in DEPC-treated water and stored at −70 °C until further use.

### RNA amplification and sequencing library preparation

Concentration and quality of the total extracted RNA was checked by using the ‘Quant-it ribogreen RNA assay’ (Life Technologies, Grand island, NY, US) and the RNA 6000 pico chip (Agilent Technologies, Santa Clara, CA, USA), respectively. One µg of RNA was used to start the library preparation. Ribosomal RNA was removed from the total RNA using the ‘Ribo-zero Magnetic Bacteria kit’ (Epicentre), according to manufacturer’s protocol. Subsequently, RNA cleanup was performed with the ‘Rneasy MinElute Cleanup kit’ (Qiagen). After fragmentation and random priming, first and second strand cDNA was synthesized using the ‘NEBNextUltra Directional RNA Library Prep Kit for Illumina’ (New England Biolabs), according to manufacturer’s protocol. After adapter ligation, the library was cleaned up twice using the Agencourt AMPure XP system (Beckman Coulter Inc.), followed by a 14 cycles PCR amplification, according to manufacturer’s instructions. Then, the library underwent a final cleanup using the Agencourt AMPure XP system (Beckman Coulter Inc.) after which the libraries’ size distribution and quality was assessed using a high sensitivity DNA chip (Agilent Technologies). Libraries were subsequently quantified by qPCR, according to Illumina’s protocol ‘Sequencing Library qPCR Quantification protocol guide’, version February 2011. Finally, equimolar quantities (1.9 pM) of all libraries were sequenced by a high throughput run on the Illumina NextSeq. 500 sequencer using 2 × 150 bp paired-end reads and a 1% Phix spike-in control.

### Read alignment and differential gene-expression analysis

A custom.gff annotation file was made for reference genome NC_000962.3 of *Mtb* H37Rv. The original RefSeq annotation file was appended with all sRNA candidate and IGR’s described in the Supplementary data Miotto *et al*., 2012 and Rodriguez *et al*., 2014. Annotations on the same strand with more than 50% overlap with another annotation were considered duplicates. After filtering by origin, where RefSeq annotations were retained over Rodriguez *et al*., which in turn were retained over Miotto *et al*., a single duplicate with maximal length remained. All annotated features: genes, IGRs and candidates were annotated as genes for gene expression analysis.

To quantify gene expression, the RNA-seq Analysis tool from the CLC Genomics Workbench 9.0.1 software (CLC Bio, Aarhus, Denmark) was used to map the reads to gene regions only with strand-specificity set to reverse and default settings. Count tables were made by counting the reads that mapped against the genes and exported from CLC.

To explore if the samples from different treatment groups clustered together and to detect outlier samples, a Principal Component Analysis (PCA) was performed in R on the rlog^49^ normalized and transformed counts of the genes expressed at cpm above 1 in at least 2 samples.

Differential gene expression analysis was performed using edgeR^50^. Genes were only retained if they were expressed at a cpm above 1 in at least 2 samples. Four different models were built. The first general linear model was made with 2 treatments “dextrose” vs “lipid”, defining the effects from the growth phases “exponential”, “stationary” and “NRP1” as batch effect. The last three general linear models were made with 2 treatments each “dextrose exponential” vs “lipid exponential”, “dextrose stationary” vs “lipid stationary” and “dextrose NRP1” vs “lipid NRP1”. A differential functional categories analysis was done for the mentioned four comparisons, using dextrose groups as control, lipids as treatment and growth stages as batch effect. Likewise, a differential pathways analysis was performed using KEGG^30^ for the general linear model D vs L.

The set of genes with significant increased expression obtained from the first general linear model (DGE: D vs L), that overlapped with significant increased expression in the subsequent DGE lineal models per stage of growth ([DE vs LE], [DS vs LS] and [DNRP1 vs LNRP1]) were grouped in a Venn diagram. This set of genes was defined as the “*core lipid response*”. Additionally, only those genes that overlapped in the three stages of *Mtb*, were called “*the mean core lipid response*”.

The data is available at the GEO database of NCBI. The accession number is GSE100097.

### qRT-PCR

The total transcripts of selected genes were measured by real-time qRT-PCR. cDNA was synthetized from 1 µg of total RNA using the “SuperScript^TM^ First-Strand Synthesis System for RT-PCR” kit (Invitrogen). Quantification was performed with gene-specific primers (see Supplemental Material Table S10), which were designed by the Primer-Blast tool^51^ and based on the genome sequence for *Mtb* H37Rv with Gene bank access number NC_000962.3. qPCR was performed by using the start SYBR Green I Master mix and the LightCycler® 480 system (Life Science Roche). Quantification was carried out in the six experimental conditions (DE, LE, DS, LS, DNRP1, LNRP1), using four technical replicates for each two biological replicates. The qPCR was subjected to 40 cycles of amplification and a melting curve analysis was performed for ensuring that the fluorescence levels detected were due to the amplification of a specific product. To determine the absolute gene expression, a standard curve was obtained for each set of primers by using 10-fold dilutions of known amounts of *Mtb* H37RV chromosomal DNA (100, 1 000, 10 000, 100 000, and 1 000 000 theoretical copies). Threshold cycle values of each RNA quantitation were interpolated to standard curve to obtain gene expression (number of gene copies per microgram of RNA). Normalization of these data was performed by using *rrs* gene (16 S rRNA) expression levels. The statistical analysis was carried out in SIGMASTAT software version 3.5, through a Two-way ANOVA and Turkey’s multiple-comparison, P value < 0.05 was considered significant.

### Statistical analysis

Statistical testing for differential gene expression (DGE) was done using the empirical Bayes quasi-likelihood F-test. For all DGE analysis performed in this work, genes, functional categories and KEGG pathways, having a false discovery rate (FDR) <0.05 and a fold change >2 were considered significantly differential. PCA was performed in R on rlog^49^ transformed cpm of the differentially expressed genes. To produce the heat map^52^, the normalized count data of the differentially expressed genes was rescaled between −3 and 3 and clustered based on Pearson correlation.

The statistical analysis for qRT-PCR results was carried out in SIGMASTAT software version 3.5, through a Two-way ANOVA and Turkey’s multiple-comparison, to determine the significance of differences of gene expression between lipid and dextrose conditions. *P* value < 0.05 was considered significant, with one degree of freedom.

## Electronic supplementary material

Supplementary Table S1.

Supplementary Table S2.

Supplementary Tables S3-S10
