# Supplementary material for: The transcriptome of Mycobacterium tuberculosis in a lipid-rich dormancy model through RNAseq analysis
Source: Sci Rep. 2017 Dec 15;7:17665. doi: 10.1038/s41598-017-17751-x (PMC5732278; doi:10.1038/s41598-017-17751-x)
Supplement: Supplementary file 3 — Supplementary Tables S3-S10 [file 41598_2017_17751_MOESM3_ESM.pdf]

SUPPLEMENTAL MATERIAL

Table S3. RNA-seq mapping results. Total number of reads (in millions) and percentages of total paired reads.

Sample	Total paired reads	Sense reads: CDS and ncRNA	Sense reads: only ncRNA	Anti-sense reads
**DE**	51.2	38.6 (75.4 %)	6.4 (12.5 %)	2.1 (4.2 %)
**DE**	49.5	36.9 (74.5 %)	5.8 (11.6 %)	2.0 (4.1 %)
**DS**	51.4	36.0 (70.0 %)	12.1 (23.5 %)	4.4 (8.5 %)
**DS**	62.7	45.1 (72.0 %)	14.3 (22.8 %)	5.9 (9.4 %)
**DNRP1**	61.8	43.6 (70.7 %)	7.9 (12.8 %)	3.6 (5.8 %)
**DNRP1**	31.0	23.4 (75.6 %)	6.7 (21.5 %)	1.3 (4.3 %)
**LE**	58.5	47.2 (80.6 %)	8.8 (15.1 %)	1.8 (3.0 %)
**LE**	53.2	42.4 (79.6 %)	9.6 (18.1 %)	1.5 (2.7 %)
**LS**	57.5	46.1 (80.1 %)	18.9 (32.9 %)	0.87 (1.5 %)
**LS**	51.7	40.6 (78.5 %)	16.3 (31.4 %)	0.94 (1.8 %)
**LNRP1**	65.8	51.1 (77.6 %)	13.2 (20.0 %)	2.6 (4.0 %)
**LNRP1**	63.7	51.4 (80.6 %)	15.7 (24.6 %)	1.6 (2.6 %)

Table S4. Differential expression analysis of functional categories. Dextrose conditions were used as control group and Lipid as treatment (general D vs L), growth stages were used as a batch effect.

Increased expression in the presence of lipids:				
Functional category	**Mean log_2 fold change**	***p* value**	**FDR**	**Set size**
Intermediary metabolism and respiration	3.2549851	0.000577347	0.005773469	920
Lipid metabolism	2.63201945	0.004369886	0.021849431	268
Regulatory proteins	0.938987183	0.174168298	0.580560994	195
Virulence, detoxification and adaptation	0.654360315	0.256602683	0.641506708	236
Cell wall and cell processes	0.382127259	0.351210219	0.702420439	761
Conserved hypothetical	0.160041507	0.436432065	0.727386776	1027
Information pathways	-0.260140305	0.602561252	0.860801789	241
Stable RNA	-0.797625025	0.785588402	0.981985503	29
PE/PPE	-2.414969445	0.991853302	0.99886261	162
Insertion seqs and phages	-3.098665085	0.99886261	0.99886261	87
Diminished expression in the presence of lipids:				
Functional category	**Mean log_2 fold change**	***p* value**	**FDR**	**Set size**
Insertion seqs and phages	-3.098665085	0.00113739	0.011373897	87
PE/PPE	-2.414969445	0.008146698	0.040733489	162
Stable RNA	-0.797625025	0.214411598	0.714705326	29
Information pathways	-0.260140305	0.397438748	0.99359687	241
Conserved hypothetical	0.160041507	0.563567935	0.999422653	1027
Cell wall and cell processes	0.382127259	0.648789781	0.999422653	761
Virulence, detoxification and adaptation	0.654360315	0.743397317	0.999422653	236
Regulatory proteins	0.938987183	0.825831702	0.999422653	195
Lipid metabolism	2.63201945	0.995630114	0.999422653	268
Intermediary metabolism and respiration	3.2549851	0.999422653	0.999422653	920

Table S5. Differential expression analysis of KEGG pathways. Differential pathways analysis with Dextrose as control group and Lipid as treatment, growth stages were used as a batch effect.

**Significant up-regulated pathways**	**FDR**	**Set size**
mtu01120 Microbial metabolism in diverse environments	0.002487853	216
mtu00984 Steroid degradation	0.002487853	13
mtu02020 Two-component system	0.03991373	54
mtu00620 Pyruvate metabolism	0.044056934	47
mtu01200 Carbon metabolism	0.044056934	111
mtu00071 Fatty acid degradation	0.044056934	50
mtu00310 Lysine degradation	0.044056934	38
mtu00280 Valine, leucine and isoleucine degradation	0.044056934	56
mtu00380 Tryptophan metabolism	0.044056934	42
mtu00650 Butanoate metabolism	0.044056934	55
mtu00190 Oxidative phosphorylation	0.044056934	46

Table S6. Differential expression analysis of functional categories for exponential stage. Dextrose exponential condition was used as control and lipid exponential as treatment (DE vs LE).

Functional category	Mean log_2 fold change	*p* value	FDR	Set size
Increased expression in LE:				
Functional category	**Mean log_2 fold change**	***p* value**	**FDR**	**Set size**
PE/PPE	1.351029443	0.088830099	0.79256419	160
Stable RNA	0.58858318	0.279661374	0.79256419	22
Regulatory proteins	0.492998416	0.311147797	0.79256419	195
Conserved hypothetical	0.476105649	0.317025676	0.79256419	998
Intermediary metabolism and respiration	-0.139092353	0.555303673	0.999254255	917
Insertion seqs and phages	-0.550710317	0.708708303	0.999254255	83
Virulence, detoxification and adaptation	-0.614619739	0.730442291	0.999254255	232
Lipid metabolism	-0.856270429	0.803878397	0.999254255	268
Cell wall and cell processes	-1.725681159	0.957695104	0.999254255	755
Information pathways	-3.196667552	0.999254255	0.999254255	241
Diminished expression in LE:				
Functional category	**Mean log_2 fold change**	***p* value**	**FDR**	**Set size**
Information pathways	-3.196667552	0.000745745	0.007457445	241
Cell wall and cell processes	-1.725681159	0.042304896	0.211524482	755
Lipid metabolism	-0.856270429	0.196121603	0.582583394	268
Virulence, detoxification and adaptation	-0.614619739	0.269557709	0.582583394	232
Insertion seqs and phages	-0.550710317	0.291291697	0.582583394	83
Intermediary metabolism and respiration	-0.139092353	0.444696327	0.741160544	917
Conserved hypothetical	0.476105649	0.682974324	0.800376251	998
Regulatory proteins	0.492998416	0.688852203	0.800376251	195
Stable RNA	0.58858318	0.720338626	0.800376251	22
PE/PPE	1.351029443	0.911169901	0.911169901	160

Table S7. Differential expression analysis of functional categories for stationary stage. Dextrose stationary condition was used as control and lipid stationary as treatment (DS vs LS).

Functional category	Mean log_2 fold change	*p* value	FDR	Set size
Increased expression in LS:				
Functional category	**Mean log_2 fold change**	***p* value**	**FDR**	**Set size**
Intermediary metabolism and respiration	3.168922275	0.000777753	0.007777532	920
Cell wall and cell processes	2.610061912	0.004571528	0.019282174	756
Information pathways	2.51481911	0.006131746	0.019282174	241
Lipid metabolism	2.430551828	0.00771287	0.019282174	267
Conserved hypothetical	-0.411979103	0.659800886	0.999974615	1017
Stable RNA	-1.091112442	0.859640497	0.999974615	26
Virulence, detoxification and adaptation	-1.29289663	0.901653686	0.999974615	233
Regulatory proteins	-1.843877562	0.967013669	0.999974615	195
PE/PPE	-2.691080125	0.996251728	0.999974615	161
Insertion seqs and phages	-4.164218156	0.999974615	0.999974615	87
Diminished expression in LS:				
Functional category	**Mean log_2 fold change**	***p* value**	**FDR**	**Set size**
Insertion seqs and phages	-4.164218156	2.54E-05	0.000253852	87
PE/PPE	-2.691080125	0.003748272	0.018741361	161
Regulatory proteins	-1.843877562	0.032986331	0.109954437	195
Virulence, detoxification and adaptation	-1.29289663	0.098346314	0.245865784	233
Stable RNA	-1.091112442	0.140359503	0.280719007	26
Conserved hypothetical	-0.411979103	0.340199114	0.566998524	1017
Lipid metabolism	2.430551828	0.99228713	0.999222247	267
Information pathways	2.51481911	0.993868254	0.999222247	241
Cell wall and cell processes	2.610061912	0.995428472	0.999222247	756
Intermediary metabolism and respiration	3.168922275	0.999222247	0.999222247	920

Table S8. Differential expression analysis of functional categories for NRP1 stage. Dextrose NRP1 condition was used as control and lipid NRP1 as treatment (DNRP1 vs LNRP1).

Increased expression in LNRP1:				
Functional category	**Mean log_2 fold change**	***p* value**	**FDR**	**Set size**
Virulence, detoxification and adaptation	4.273008619	1.18E-05	0.000117511	233
Regulatory proteins	3.925030603	5.17E-05	0.000258685	195
Lipid metabolism	2.820063698	0.00249043	0.008301433	267
Intermediary metabolism and respiration	2.309445168	0.010514734	0.026286835	920
Conserved hypothetical	0.368607862	0.35622953	0.712459059	1002
Insertion seqs and phages	-0.101260223	0.540264856	0.900441427	84
Stable RNA	-0.399475572	0.654342281	0.934774687	25
Information pathways	-1.44441487	0.925312568	0.998612354	240
Cell wall and cell processes	-1.642080768	0.949606567	0.998612354	756
PE/PPE	-3.015187087	0.998612354	0.998612354	162
Diminished expression in LNRP1:				
Functional category	**Mean log_2 fold change**	***p* value**	**FDR**	**Set size**
PE/PPE	-3.015187087	0.001387646	0.013876457	162
Cell wall and cell processes	-1.642080768	0.050393433	0.248958105	756
Information pathways	-1.44441487	0.074687432	0.248958105	240
Stable RNA	-0.399475572	0.345657719	0.864144298	25
Insertion seqs and phages	-0.101260223	0.459735144	0.919470288	84
Conserved hypothetical	0.368607862	0.64377047	0.999988249	1002
Intermediary metabolism and respiration	2.309445168	0.989485266	0.999988249	920
Lipid metabolism	2.820063698	0.99750957	0.999988249	267
Regulatory proteins	3.925030603	0.999948263	0.999988249	195
Virulence, detoxification and adaptation	4.273008619	0.999988249	0.999988249	233

Table S9. Quantification and statistical analysis of gene expression determined by qRT-PCR.

GENE	STAGE	Dextrose conditions			Lipid conditions			STATISTIC ANALYSIS Two Way-ANOVA between: dextrose vs lipids				Relative expression. Fold change*
		Mean	Std Dev	N	Mean	Std Dev	N	DF	Mean Square	F Ratio	*P* value	
rv3160c	Exponential	1.6E+05	3.6E+04	6	1.5E+06	5.9E+05	8	1	5.9E+12	2.8E+01	<0.001	9.4↑
	Stationary	2.1E+05	1.3E+05	7	1.1E+06	3.0E+05	3	1	1.5E+12	4.5E+01	<0.001	5.1↑
	NRP1	7.8E+04	3.3E+03	4	2.5E+07	2.0E+07	7	1	1.6E+15	6.4E+00	0.033	326.8↑
rv3161c	Exponential	1.3E+03	1.6E+02	3	1.1E+05	1.3E+04	4	1	2.0E+10	1.9E+02	<0.001	82.7↑
	Stationary	6.7E+04	4.7E+04	7	4.8E+05	1.3E+05	3	1	3.5E+11	5.9E+01	<0.001	7.1↑
	NRP1	1.5E+04	6.6E+03	7	5.6E+06	5.1E+06	7	1	1.1E+14	8.4E+00	0.013	372.2↑
PPE53	Exponential	2.1E+05	6.7E+04	7	5.1E+05	1.1E+05	6	1	2.9E+11	3.4E+01	<0.001	2.4↑
	Stationary	5.3E+04	8.0E+03	3	5.8E+04	1.0E+04	3	1	3.5E+07	4.2E-01	**0.553**	1.1
	NRP1	2.9E+05	2.1E+05	6	5.6E+06	4.3E+06	6	1	2.9E+11	3.4E+01	<0.001	19.1↑
che1	Exponential	6.0E+05	7.7E+04	6	9.6E+05	4.7E+05	7	1	4.4E+11	3.5E+00	0.089	1.6↑
	Stationary	5.0E+04	1.9E+04	6	9.0E+05	1.3E+05	3	1	1.4E+12	2.6E+02	<0.001	17.9↑
	NRP1	1.3E+05	2.7E+04	3	2.7E+05	2.4E+04	4	1	3.5E+10	5.6E+01	<0.001	2.1↑
mmpS5	Exponential	1.4E+05	5.4E+04	8	5.9E+06	1.9E+06	6	1	1.1E+14	7.6E+01	<0.001	41.4↑
	Stationary	7.4E+04	8.9E+03	4	1.6E+06	3.3E+04	4	1	4.9E+12	8.5E+03	<0.001	22.0↑
	NRP1	1.9E+06	8.0E+05	8	1.5E+07	1.3E+07	6	1	5.7E+14	7.6E+00	0.017	7.9↑
hsd4A	Exponential	4.8E+03	6.9E+02	3	2.0E+04	9.1E+03	4	1	3.9E+08	7.7E+00	0.039	4.2↑
	Stationary	3.4E+04	7.5E+03	6	4.6E+05	1.1E+05	3	1	3.6E+11	1.0E+02	<0.001	13.4↑
	NRP1	1.5E+05	4.3E+04	6	1.1E+06	9.8E+05	6	1	2.6E+12	5.4E+00	0.043	7.0↑
usfY	Exponential	9.1E+04	2.6E+04	6	1.1E+06	4.2E+05	6	1	2.9E+12	3.3E+01	<0.001	11.9↑
	Stationary	1.1E+05	5.3E+03	3	2.9E+06	2.9E+05	3	1	1.2E+13	2.8E+02	<0.001	26.7↑
	NRP1	1.5E+06	9.2E+05	6	2.5E+07	2.4E+07	7	1	1.9E+15	6.1E+00	0.031	16.8↑
hrp1	Exponential	3.4E+05	8.5E+04	6	2.6E+07	1.7E+07	7	1	2.1E+15	1.4E+01	0.003	76.4↑
	Stationary	4.0E+05	1.2E+05	6	3.1E+06	4.0E+05	3	1	1.4E+13	2.5E+02	<0.001	7.6↑
	NRP1	2.1E+07	9.8E+06	7	1.7E+07	2.6E+07	6	1	1.1E+15	5.2E+00	**0.051**	-1.2
Rv1639c	Exponential	4.0E+05	1.8E+05	7	3.1E+05	7.4E+04	6	1	2.7E+10	1.3E+00	0.274	-1.3↓
	Stationary	1.1E+04	2.8E+03	6	7.2E+04	1.4E+04	3	1	7.5E+09	1.2E+02	<0.001	6.4↑
	NRP1	5.0E+05	2.7E+05	6	4.6E+04	1.1E+04	7	1	6.6E+11	2.0E+01	0.001	-10.8
Rv0560	Exponential	9.2E+04	1.7E+04	7	5.9E+04	9.2E+03	7	1	3.7E+09	2.0E+01	<0.001	0.6↑
	Stationary	1.6E+05	1.5E+05	7	4.9E+04	6.2E+03	4	1	3.4E+10	2.1E+00	0.177	-3.4↓
	NRP1	6.2E+04	4.7E+04	7	9.2E+06	6.0E+06	7	1	2.9E+14	1.6E+01	0.002	148.3↑

*Ratio of gene expression in lipid/dextrose conditions. Increased (↑) and diminished (↓) expression in lipids.

Table S10. Primers used for qPCR

Gen / Rv number	Primers	Product name	Design reference
rpsL / rv0682	Fw: 5’-CCTTCAGAGCCGCGGTCT- 3’<br>Rev: 5’-CCAACCATCCAGCAGCTGG- 3’	Ribosomal protein S12	1
rrs / MTB000019	Fw: ATGACGGCCTTCGGGTTGTAA<br>Rev: CGGCTGCTGGCACGTAGTTG	Ribosomal RNA 16S	1
rv3160c	Fw: CTGGACCACCTGCATGCTTC<br>Rev: CCATCTCCGCGACTAGCTCC	Possible transcriptional regulatory protein (probably TetR-family)	2
Rv3161c	Fw: CAGGGTTCCCTTCACCGTTC<br>Rev: GCGGAATAAAGCCGAACCAC	Possible dioxygenase	2
PPE53 / rv3159c	Fw: CAATGCCCACCAAGTTGTCG<br>Rev: ACATCGGCTCCTCCAACATC	PPE family protein PPE53	This study
che1 / rv2393	Fw: 5’ -GATCGGCTCATCGCATCCC- 3’<br>Rev: 5’ -CTCCGGTCGGGTGATGAAAG- 3’	Ferrochelatase Che1	This study
mmpS5 / Rv0677c	Fw: GCCGGAAACTTCGTACTCCA<br>Rev: CCGCACTTTCTTTGGTTCCG	Possible conserved membrane protein MmpS5	This study
hsd4A / rv3502c	Fw: 5’ -TTGCGGGTGAGCAGAAAATG- 3’<br>Rev: 5’ -CATCGTGGTGAACAATGCCG- 3’	Possible 17-beta-hydroxysteroid dehydrogenase	This study
usfY / rv3288c	Fw: 5’ -ACAGATACCCAGGCCAATGC- 3’<br>Rev: 5’ -CGGAATAGGACGGAAACGGA- 3’	Putative protein UsfY	This study
hrp1 / rv2626c	Fw: GCGGACCTGATGTTCTTCCA<br>Rev: CTAGACCCGAATACCGCCAC	Hypoxic response protein 1 Hrp1	This study
rv1639c	Fw: ATCCACGAATACCACGACGG<br>Rev: GTCAATTCAACACACCCGCC	Conserved hypothetical membrane protein	This study
rv0560	Fw: CGATTGCAGATAGCCCTCCC<br>Rev: TTTACCGGCTATGACGGCAG	Possible benzoquinone methyltransferase (methylase)	This study

REFERENCES:

1. Badillo-López, C. *et al.* Differential expression of dnaA and dosR genes among members of the Mycobacterium tuberculosis complex under oxic and hypoxic conditions. *Int. Microbiol.* **13,** 9–13 (2010).
2. Betts, J. C. *et al.* Signature gene expression profiles discrimate between isoniazid- thiolactomycin-, and triclosan-treated Mycobacterium tuberculosis . *Antimicrob.Agents Chemother.* **47,** 2903–2913 (2003).
